# Navigating the journey of Aboriginal childhood disability: a qualitative study of carers’ interface with services

**DOI:** 10.1186/s12913-016-1926-0

**Published:** 2016-12-01

**Authors:** Anna Green, Penelope Abbott, Patricia Delaney, Patrick Patradoon-Ho, John Delaney, Patricia Mary Davidson, Michelle DiGiacomo

**Affiliations:** 1 University of Technology Sydney, Center for Cardiovascular and Chronic Care, Faculty of Health, PO Box 123, Broadway, Sydney, NSW 2007 Australia; 2 Western Sydney University, Locked Bag 1797, Penrith, NSW 1797 Australia; 3 Western Sydney University, Blacktown Mt Druitt Hospital, Western Sydney Local Health District, Blacktown Road, Blacktown, NSW 2148 Australia; 4 University of Technology Sydney, Center for Cardiovascular and Chronic Care, Faculty of Health, Johns Hopkins University, School of Nursing, 525 N. Wolfe Street, Baltimore, MD 21205 USA

**Keywords:** Aboriginal and Torres Strait Islander, Childhood, Disability, Carer, Service pathway

## Abstract

**Background:**

The disadvantage experienced by Aboriginal and Torres Strait Islander children with a disability is well recognized. The long term consequences of failing to address disability on health, education and employment underlies the importance of early intervention. Caregivers experience a disproportionate burden and have challenges accessing services. The aim of this study was to describe the carer journey of accessing support and services.

**Methods:**

We conducted in-depth semi-structured interviews with nineteen parents and carers of Aboriginal children aged 0–8 years. The children were patients at a child developmental clinic at a metropolitan area Aboriginal health service in Eastern Australia. Interpretive phenomenological analysis was applied to transcribed verbatim accounts.

**Results:**

Four themes were developed using the ‘journey’ metaphor to describe the carer pathway of accessing support and services at the community, service and policy levels. Themes included 1) the need for increased signage within communities via community education, information and awareness, 2) wrong way signs, roundabouts and roadblocks encountered when accessing services, 3) alternate routes can facilitate the journey, and 4) incompatibility of inflexible bureaucratic road rules and lived realities.

**Conclusions:**

The challenges of caring for a child with a disability are indisputable and these can be compounded for people experiencing socio-economic disadvantage and marginalisation. Overcoming challenges to service access faced by carers of Aboriginal children with a disability will require investment in community, services and policy to tailor culturally appropriate models of care.

**Electronic supplementary material:**

The online version of this article (doi:10.1186/s12913-016-1926-0) contains supplementary material, which is available to authorized users.

## Background

### Disparity in Aboriginal and Torres Strait Islander childhood disability - impact on outcomes

The opportunity for health, social development, education and wellbeing in people with disabilities can be easily blighted by adverse social and environmental forces and commonly the needs of individuals can be invisible [1]. In Australia, Aboriginal and Torres Strait Islander peoples suffer worse health and more disadvantage than other Australians, the effects of which are exacerbated by dispossession, disempowerment and racism [2, 3]. The disparities between Aboriginal and Torres Strait Islander children and other Australian children are also highlighted in experiences of disability [4–6]. Aboriginal and Torres Strait Islander children are more likely to experience hearing loss [7–9], linked to higher rates of middle ear disease such as otitis media [4], and to require assistance with self-care, mobility or communication than other children [10]. Disparities have also been reported in the prevalence of developmental delay [11].

The high prevalence of disability can have life-long negative consequences on health, education and employment outcomes for Aboriginal and Torres Strait Islander children [9, 12]. Evidence links low educational achievement to involvement in the criminal justice system [13]. On average Aboriginal and Torres Strait Islander youth are detained within the juvenile justice system at 24 times the rate of other youth [6] and are 4 to 5 times more likely to have an intellectual disability than the general population [14]. Adolescents coming into contact with the juvenile justice system are more likely to be incarcerated as an adult [15].

The World Health Organization recognizes the importance of social determinants in influencing health outcomes, and the Close the Gap Campaign is an important focus of Australian government and non-government organisations to address the needs of Aboriginal and Torres Strait Islander people. Internationally, indigenous children with a disability are considered ‘doubly disadvantaged’ [16, 17]. A recent longitudinal cohort study of development in urban Aboriginal children found that high levels of socio-economic disadvantage are a predictor for developmental progress [18]. While the high levels of socio-economic disadvantage increases the chance of having a disability, the high rates of disability can contribute to socio-economic disadvantage, thus reinforcing a life-long cycle [6].

### Impact of service access barriers on early intervention

Early intervention is crucial to counteracting the negative impact of disability for Aboriginal and Torres Strait Islander children [4]. It is necessary to facilitate timely access of children and their families to appropriate health services, social support services and treatment. This involves interacting with a health care system that is not always accommodating of unique, socio-cultural needs. Involvement of sectors other than health, such as education and social services, is also required for effective early intervention but increases the complexity of engagement [18–20]. Carers report a number of barriers to accessing early intervention and support services [21]. Lack of awareness of appropriate disability services, frequent absence of culturally appropriate support, insufficient resources to provide care, and a loss of social-capital-based support due to fractured family and community structures have been reported as barriers to service access which impedes early intervention [21, 22].

### A holistic approach to service access

Improving service access is important to addressing health disparities [23]. Influencing factors exist at the micro, meso and macro levels [24–27]. A holistic approach is required when addressing service access issues for Aboriginal and Torres Strait Islander families of a child with a disability [25, 28]. Desirable outcomes are unlikely to be achieved if the interdependency of influencing factors is not addressed. For example, carers play a central role as navigators of their child’s care [29], thus, exploring the experiences of carers is imperative in understanding service access for children with a disability.

Data on Aboriginal and Torres Strait Islander children with a disability are limited, particularly in urban populations and this inhibits adequate service planning [30]. Although over half of the Aboriginal and Torres Strait Islander population live in urban or regional areas, most of the research on Aboriginal and Torres Strait Islander childhood disability is on rural or remote populations [24, 31–33]. It is important to address this gap in knowledge as Aboriginal and Torres Strait Islander populations can be less visible in urban areas and available services are not necessarily appropriate or accessible [21, 32, 34, 35]. To address this paucity of information, we investigated the experiences of Aboriginal carers to inform service planning and access to early intervention.

## Methods

We aimed to describe the carer experience of accessing support and services and investigate the barriers and facilitators to service access from the perspective of carers. We used a socio-ecological framework to situate carers’ experiences at the macro- (government), exo- (organizational) and meso- (provider/community) system levels in recognition of the interaction and inter-dependence of these environmental factors. The carer, child and family are positioned within the centre, or the individual-level, of the socio-ecological framework. The micro-level (individual) experiences included caring for the child and family, challenges and facilitators to this caring, carer health and wellbeing and associated financial expenses and non-economic costs (manuscript under review). This paper reports on the carers’ interaction with systems beyond their immediate family; that being, the community, service and policy levels of the framework in pursuit of optimal outcomes for their child with a disability. To understand carers’ experiences and perspectives of seeking and obtaining health and social service supports for their children, we used a phenomenological approach [36].

### Context, participants and recruitment

Participants were parents or informal primary carers (hereafter, carers) of children aged 0–8 years who were patients at a child development clinic at a metropolitan area Aboriginal health service. The clinic caters to children with developmental problems aged from birth to 16 years and deals with the identification and management of conditions such as attention deficit hyperactivity disorder, behavioural problems and developmental delays. Clinic staff informed potential participants about the study and supplied information sheets and flyers to interested parties. The term disability was deliberately kept broad to include a range of disability experiences including mild, moderate and severe, involving physical, intellectual or developmental issues. Recruitment continued until no new issues emerged.

### Data collection

The research team was co-led by Aboriginal and non-Aboriginal team members, each with designated tasks and expertise, who frequently met to discuss research processes, debrief about ongoing data collection, and strategize to overcome logistical challenges. To capture the range of experiences and perspectives of caring for a child with a disability, we conducted in-depth semi-structured interviews at two time-points with each participant. Initial interviews were conducted at the health facility in private clinic rooms with one of two researchers trained in qualitative methods (AA & BB). These interviews lasted approximately 20–60 min and often occurred opportunistically while waiting for or immediately following their child’s clinic appointment. Children sometimes were in the room during interviews, usually playing or sleeping, because no other supervision was available.

Follow-up interviews were conducted with participants to ascertain and document activities related to the child’s disability, services sought, interactions with health or other service professionals, and barriers and facilitators to support since the first interview. Follow-up interviews took place via telephone if participants were unable to attend the service in person. All follow-up interviews were conducted within 6–12 months of the initial interviews according to the preference and availability of participants. Participants were reimbursed for travel and child care expenses incurred as a result of participation. Data collection took place from April 2013 to June 2015.

### Interviewers

The Aboriginal leaders and cultural mentors of the project were unable to undertake interviews given their management roles at the health facility. This meant that the non-Aboriginal team members, who worked in another area of the health service one or more days each week, were designated to conduct the interviews whenever clinic staff telephoned with an eligible consenting participant. One of these interviewers was a general practitioner (GP) at the health service and a university academic. She had met some of the participants previously in the context of primary care consultations, however she was not involved directly in the child’s healthcare or the clinic. Prior to commencing interviews, she explained the research role to participants as separate to her GP role. As a non-Aboriginal woman having worked part-time for over twenty years at the health service where this research was conducted, this interviewer had extensive knowledge and experience within the local community and health service. The second interviewer, also a non-Aboriginal woman, had qualifications in psychology and was a health services researcher based at a university. She had been a weekly visiting counsellor at the health service for 8 years.

Two local Aboriginal elders (CC & DD) co-led this project and acted as cultural mentors who contributed to the study design, recruitment and analysis. At each stage, they offered perspectives of findings conveyed in repeated discussions with the interviewers. Each described their positioning in relation to this research and described their conscious biases to clarify their interpretations. Some, but not all, members of this team had the experience of parenting or caring for a child with a disability. The interviewers met periodically to debrief and consider interview proceedings. Each provided important insights that informed the others’ interpretations. The cultural mentors and GP interviewer provided service and community context from Aboriginal and non-Aboriginal perspectives, respectively, while the university interviewer offered a less entrenched experience of the community, yet with an appreciation of historical and social context.

The semi-structured interviews attended primarily to the carer’s own narrative. Interview topics were derived from a literature review [24], experience and expertise of cultural mentors who were also carers, and the social determinants of health and social capital frameworks [2, 17, 37] (Additional file 1). During interviews, we explored participant’s experiences with the child’s disability, their meanings and interpretations of these experiences, and their experiences seeking and obtaining support and services. Attention was paid to experiences of intake and triage, respite use and need, allied health service access and needs and preferences for information, services and support. Limited demographic and health-related information was collected during the interviews. Upon the follow-up interview, the interviewer summarised thematic content of the previous interview with participants to seek confirmation of its validity [38]. Any noted discrepancies were discussed, clarified and resolved to the satisfaction of the participant.

### Data analysis and trustworthiness

Analysis began with development of a contact summary sheet with key demographic information and emergent issues [39] for each interview. Interpretative phenomenological analysis (IPA) was used to analyse carers’ experiences [36] as this approach centres on individuals ascribing meaning to their experiences in their interactions with the environment. IPA is a set of systematic processes that shift from phenomenological to interpretive while focusing on the participant’s perspective and understandings in different contexts [36]. It is an iterative, inductive and flexible approach involving close reading and re-reading of transcripts while note-taking in margins, ‘bracketing’ the analyst’s critical perspective, recording critical and interpretive comments in a reflexive diary; re-reading the text and identifying codes and themes that best capture the essential qualities of that interview while also looking for connections between themes; revisiting earlier transcripts to re-consider data; clustering themes and concepts and developing an overall structure using excerpts from interviews; and re-assessing and revising new themes against earlier data [40]. One researcher (AA) undertook preliminary analysis wherein transcripts were read, notes added to the document, and a coding system developed to elucidate categories. To facilitate rigor, a second researcher (EE) independently coded unmarked transcripts [41]. The interviewers had frequent informal and formal discussions regarding code development and emerging findings with the lead cultural mentor (CC) who provided formative insights throughout the data collection and preliminary analysis period; divergences in the coding scheme were discussed until consensus was reached. The remaining interviews were coded according to the developed scheme, yet emergent categories were documented and considered throughout the analysis. Categories were then collapsed into themes. Preliminary and developed themes were discussed with the full project team prior to cessation of analysis and this was followed by another extensive discussion of key themes with the lead cultural mentor. Transcript excerpts which supported each theme were copied into a Microsoft Word file along with supporting field notes. All supporting evidence was then considered, contextualised and written into an account illustrating participants’ experiences and perspectives [42].

To further enhance credibility and trustworthiness, stakeholder checks were undertaken [41], whereby preliminary analysis of findings was presented to clinic staff, some of whom were also carers of an Aboriginal child with a disability. Additional feedback from this group was incorporated into theme development.

### Ethical considerations

Ethical approval was granted by the Aboriginal Health and Medical Research Council (AH&MRC) (762/10) and University of Technology Sydney Human Research Ethics Committee (UTS HREC 2011-417R). The study adhered to key principles for research with Aboriginal and Torres Strait Islander peoples as espoused by the AH&MRC. Briefly, these included the involvement in and control of all research stages by the Aboriginal health service, direct consultation with members of the community affected by the research, reimbursement of expenses associated with participation, and intended outcomes that inform the design and delivery of needed culturally appropriate services for children and their families and are aimed at increasing the community’s knowledge of and ways to access support and services [43]. Confidentiality of discussions was assured and participants were advised that their care or status at the health service would be influenced neither by participation nor divulgence. Permission to audio record interviews was obtained from each participant. Names were replaced with pseudonyms and identifying information was removed following verbatim transcription of recordings. Study findings are reported according to the consolidated criteria for reporting qualitative research guidelines [44].

### Availability of data and materials

The data supporting the conclusions of this article are included within the article in the form of interview excerpts. Full interview transcripts remain the property of the participating Aboriginal Community Controlled Health Organization.

### The journey metaphor

Pathways and journeys have increasingly been used to depict experiences of health conditions and service use as these terms reflect movement or progression, not always in a linear direction, but generally resulting in an accumulation or loss of knowledge or other resources over time [45].

We often refer to gaining new experience or skills as ‘going on a journey’. This metaphor implies that there is a process, a starting point and a destination on which people travel, usually figuratively, in acquisition of information or resources. This is often an ongoing process that involves a series of points and opportunities for learning and this occurs over a long span of time. Some continue along a pre-determined route or course of action, while others forge a path as a result of circumstances or opportunity. The term ‘clinical pathway’ is used in health to depict standardised, evidence-based multidisciplinary management plans, of a sequence of interventions, timeframes, milestones and expected outcomes for a patient group [46]. These pathways are often depicted in resources to map the patient journey, to foster an understanding of the whole pathway (including environmental influences) and its distinct components, including specific steps or critical points along the care pathway [47]. Pathways to service delivery have likewise been depicted as roadmaps.

Although we had not anticipated, originally, use of metaphor to help us understand caregiving or care-seeking behaviour, we became aware of the symbols, words and metaphors used to convey interpretations of meaning [45]. We began the analysis process by trying to describe what we saw in experiences. Metaphors arose from our and participants’ descriptive language related to the experiences. The visual representation of these types of concepts through metaphors aligns with the narrative approach of telling stories that values the spoken word and oral history tradition in Aboriginal culture and is considered a respectful research technique [48]. Following the second stakeholder check with cultural mentors (CC & DD) this depiction was deemed a culturally congruent representation of the findings. We link the findings to this metaphor in an effort to communicate this complex phenomenon.

## Results

Participants were 19 carers of Aboriginal children. They were all women, more than half of whom (n = 10) were lone carers (without a partner or spouse), and were taking care of 60 children at home, half of whom were identified as having a disability or developmental delay. The majority of participants were the mothers of the children (n = 16), and three were grandmothers. Factors that influenced carer journeys to access support and services reflected their interactions with community, service and policy levels. Themes depicting these journeys included: *need for increased signage* *via* *community education, information and awareness; wrong way signs, roundabouts and roadblocks encountered when accessing services;* *alternate routes can facilitate the journey*; and the *incompatibility of inflexible bureaucratic road rules and lived realities*.

### Need for ‘increased signage’ within communities via community education, information and awareness

The community, defined as an informal network of extended family, friends and other carers, played a key role in carers’ accessing support and services. Due to this influence, carers emphasised the need for investment in building community capacity as a support mechanism through increased education, information and awareness.

Carers accessed advice and recommendations from community members on the developmental progress of children and available services. Some carers experienced tension between respecting community advice while knowing something is wrong with their child. Community advice in this context is inclusive of cultural advice as identified by carers. Advice by community members that there was nothing wrong with a child contributed to delays in seeking diagnoses and treatment.

*“Yeah, you let things slide. You just – it’s not that you don’t want to put the effort into it and go and sit around and take them out of school or anything like that, it’s just you’ve got your elder saying to you, “No, they’re right. They’re right. Don’t worry about it. They’ll pick up in their own time,” and sometimes they don’t.” (Rita)*

The lack of community education, information and awareness around disability and available services evidently impeded access to support and services.

*“It’s not advertised that they have this other health service that’s provided for black kids… But yeah. It’s not known…That’s what my thing is…But how many other people are missing out?” (Laura)*

Carer preferences to address this lack of clear direction and mapping were access to a local database of preferred providers and building a community of carers through support groups. Carers identified that they would prefer to have the database and support groups organised and hosted through the local Aboriginal health service.

*“I think that that would be something that would be helpful for us to just be able to have some sort of connection to other families, in particular Aboriginal families…and whether the* [Aboriginal health service] *can, sort of, do that.” (Ainslee)*

### Wrong way signs, roundabouts and roadblocks encountered when accessing services

Carers encountered a number of obstacles when attempting to access and interface with health and support services. Many carer journeys involved referrals to and interaction with multiple service providers including occupational therapists (OT), speech therapists, physiotherapists, paediatricians and GPs. In navigating this pathway independently, there were accounts of providers qualifying the extent of their service leading to inconsistent service access.

“*I would ring a private speech and private OT, and stuff and a lot of them would say, okay, but we don’t deal with children with significant disabilities… you need to go to your GP, and it would be like [laughs] a sort of a catch-22…It would just be round in circles…it just really wasn’t helpful at all.” (Ainslee)*

Delays to assessment and treatment caused by long waitlists were a significant roadblock encountered by carers. Long waitlists for needed procedures could lead to children missing school, lack of developmental progress and stress for carers.

*“And I said, I understand there is kids that are a lot worse than* [child]*. I do understand that. It’s just, with this waiting list, he’d probably be able to speak by then, he wouldn’t even need it… And then I got upset, and I said to my mum, it’s like no-one’s out there that wants to help.” (Tabitha)*

*“Waiting and waiting for appointments, you know, like, nothing gets done straight away…it’s just such a long process…I am no closer now than I was then. That’s the frustrating part about it.” (Nadia)*

Only one carer expressed lack of concern regarding waitlists stating that they are a *“part of life” (Rita)*. Contributing to this acceptance was contextualizing the carers’ own situation against the belief that there is always someone whose need is greater.

Lack of follow-up led to children falling through the treatment gap due to missed opportunities for timely access to support and services. In one case an initial needs assessment generated a list of required therapeutic interventions for one child but no follow-up contact was made to link the carer with the required services. Despite the carer following-up there had been no additional contact made with her or consolidation of these supports. Carers identified that lack of follow-up was also sometimes due to clerical errors.

“*We went and had the very first assessment where* [government service] *said, yeah, she needs OT, she needs speech, she needs physio and we never heard from them again. It was, sort of, like…they would ring us and – and, um, say that, you know, we’re still, sort of, on the waiting list…and it got to two years and we’d, sort of, had nothing…I spoke to them and they, sort of, said, well we’ll find out what’s happening and it just, sort of, never eventuated.” (Ainslee)*

*“I thought I signed everything and I thought everything got faxed through. Actually, everything got faxed through to* [children’s hospital] *and they, um, lost the paper and then it had to be re-faxed through. So, yeah and I had to re-sign all the papers. Not much – like I didn’t know much about that then because I thought everything was going ahead, going through, thought everything was fine.” (Samantha)*

Lack of assistance from service providers impeded attempts at accessing support and services. Carers described a lack of assistance in seeking funding for services such as teachers aids as well as in managing behavioural problems. This had a significant impact on the health and well-being of some carers.

*“He was more or less going to be a forgotten child and if he didn’t keep up his grades they weren’t going to keep him back but they weren’t going to give him no more help…. but it had me at breaking point where I really felt like I was having a nervous breakdown. I’ve been in tears taking him to school, bringing him home.” (Rita)*

Many participants did not have access to private transportation. Difficulty attending appointments without private transport was a prominent roadblock for carers. The majority of carers had no private means of transport which compounded difficulties associated with attending multiple appointments at multiple services. Some carers described having to begin the journey hours well in advance of appointments if referrals were made to geographically distant areas. Caring for more than one child with a disability added another layer of complexity for carers especially if the children attended separate schools.

*“So they wanted me, pick her up and then bring her here, drop her off. I said, no, no, no, no, it’s too complicated for me. Pick up the boys, you go to the school, oh no, we can’t do that.” (Helen)*

The extra cost of using taxis caused additional stress for carers. Carers who used public transport to avoid this cost experienced difficulty with managing children, especially when they required a stroller or wheelchair.

Addressing these roadblocks was particularly important in facilitating access to assessment services in order to get a diagnosis. Without a diagnosis it was difficult for carers to access support and services.

“*The school actually told me that they would not help until* [child] *did have an MRI to see if there was something wrong because I’ll quote the words of the principal, “He is like he has got a locked door and we cannot find the key to open it. Until we find the key to open it, we cannot do no more with him. We just have to leave that door locked”.” (Rita)*

For some carers, obtaining a diagnosis to access support and services was influenced by whether their child’s condition was acute or non-acute. Comparison of carer journeys suggest that children with more acute conditions fit better into the streamlined medical model of care compared to children who have non-acute conditions which are not as severe or easy to diagnose.

### Alternate routes can facilitate the journey

In the face of roadblocks, alternate routes in the form of models of care that differ to the Western bio-medical approach and supportive administrative staff and organisation procedures enabled carers to access support and services.

A model of care that viewed the needs of the child and carer holistically, provided a one-stop-shop and implemented a centralised team-based approach was identified as important by a number of carers. Many carers accessed this alternate route to support and services through local Aboriginal Community Controlled Health Organisations (ACCHOs), yet access was limited. Providing support and services through a holistic lens was essential for carers as a broad range of personal and environmental factors influence the experience of caring for a child with a disability and the ability to access support and services.

*“This is why I keep coming back here, because they were fantastic. Ah, um, not only do they help with* [child]*, they help with housing, they help with me with my ex-husband, you know what I mean. Um, they help me with getting some counselling…” (Jocelyn)*

The convenience of a one-stop-shop and centralised team-based approach was also important. At a local Aboriginal health service convenience was ensured by holding all health records in one place, linking appointments with different internal services so they were scheduled in close succession, and acting as a conduit to external services when required.

Supportive administrative staff and organisation procedures facilitated carer access to support and services. Key administrative personnel were identified as important to facilitative organisation procedures. One carer reported that a former manager used to make sure that “*everyone was doing what they were supposed to be doing” (Helen)*, however when the staff member left the organisation, this carer had difficulty reaching the service by phone. The positive impacts of organisation procedures were characterised by flexibility with payment procedures and maintaining confidentiality.

*“Our chemist is really good. Like, you know, if you’re short of cash they let you, you know, pay it next time. I mean, little things like that…they are very good down there. Very confidential…They don’t yell out… [loud voice] “Your Ritalin is ready.” Or, “Your Concerta’s ready”.” (Laura)*

### Incompatibility of inflexible bureaucratic road rules and lived realities

Policies guiding government agencies and funding bodies have far reaching effects on carer journeys to access support and services. Many carer interactions with government agencies were defined by barriers generated from inflexible rules and bureaucratic processes. Carers’ accounts indicated that the lived reality of caring for a child with a disability is not supported or acknowledged by current policy and this negatively impacted their experiences and ability to care for their child.

Carers described incidents where agencies were unable to accommodate their specific needs due to inflexible rules. Accessing financial assistance from Centrelink to assist with caregiving related responsibilities was particularly difficult due to rigid eligibility criteria. This had implications for carer employment and support payments.

*“…the Carer’s Allowance loan was supposed to be coming up, and I wanted to pay the rest of it off to get another loan for* [children’s hospital]*, because I have to pay for an overnight stay with her, and for one of these tests I have to pay for it. And they won’t be able to help me until the 27* ^*th*^ *of this month. And my appointment is on the 25* ^*th*^ *.” (Lesley)*

The Department of Housing (DoH) was another government agency many carers interacted with which was also defined by inflexible rules. Carers described DoH expectations of clients caring for a child with a disability as inappropriate. The strict requirements for obtaining housing assistance can negatively impact carers accessing support and services for their child with the lack of assistance perceived as dismissiveness.

“…*they still wanted me to look for, for pretty much three houses a day, um, um and I’ve got no personal transport…I told the Department of Housing, you know, “You are expecting me to drag my daughter around with a disability to look for houses.” They wanted – they said they wanted me to go out to* [suburb 1] *and everything. Like* [suburb 2]*, and then I said, “Like why would I go out to* [suburb 2] *when my family is in* [suburb 3]*?”…and I’m like, “Yeah, but I can’t live in* [suburb 2] *or* [suburb 1] *or anywhere I have no support,” and they were like, “Oh well, it doesn’t matter, as long as, um, you get a house”.” (Samantha)*

Bureaucratic requirements of government funding mechanisms and support services reinforce rigid eligibility criteria that mean some children don’t receive the support they require. The rigid eligibility criteria of age-based funding structures was identified as an issue influencing the quality of specialists that children with a disability have access to.

*“So yep, it doesn’t help a lot of the families that have the younger kids and that’s why - and I’ve always said that that’s why they don’t get seen to the right people because of the financial cost of that.” (Grace)*

Rigid eligibility criteria for accessible Aboriginal education officers (AEOs), who act as liaison and support workers within schools, also impacted partnerships between schools and carers. One school did not have the required percentage of Aboriginal children to qualify for a dedicated AEO. For this carer it was important to have access to the support of an AEO as they play an important support role to the carer getting across their point of view as a third party in the school-carer partnership.

*“So, yeah, it’s not feeling like you’re ganged up on, kind of thing.” (Rita)*

## Discussion

Carers’ descriptions of their attempts to access support for their child with a disability were akin to a journey: sometimes they did not know where to go (as a result of poor signage), they went around in circles, in the wrong direction, had to make u-turns and encountered roadblocks and traffic. For many who set out on a journey, they eventually arrive at a destination, but these carers were still traveling, as caring for a child with a disability is a lifelong voyage. Some carers received directional assistance along the way; people who unlocked gates and facilitated access to needed resources. For instance, the role of community both facilitated and obfuscated carers’ service access. Community influence has likewise been identified in culturally and linguistically diverse (CALD) carers wherein extended family and community members sometimes presented a barrier to access by denying presence of a disability [49]. Another Australian study of CALD carers’ perceptions of preventive health care for their children found that social influence plays a key role in identification of developmental problems and the need to access services [50]. In their review of vulnerable groups access to healthcare, Dixon-Woods et al. [51] found that people from socio-economically disadvantaged backgrounds are less likely to present for services due to the normalization of poor health within their communities and a fear of being ‘blamed’ by health care professionals [51].

Rather than not recognizing the need to get help, the ‘wait and see’ approach advised by some community members may reflect the Aboriginal world view of health [52] whereby disability is ‘part of a continuum from perfect wellbeing to death’ [32] compared to the more narrow medical definition. Gilroy et al. [53] assert that labeling individuals as disabled is offensive to some Aboriginal communities and associated with past government policies that led to removal of children from families [53]. It is also suggested that in some Aboriginal communities support and care for a person with a disability is viewed as the responsibility of family and kinship networks and outside help viewed as questioning family competence [22, 53]. Despite impacting carer access to services, the resilience and strength of Aboriginal communities in caring for each other represents a key resource that requires investment. Community capacity can be built by increasing ‘signage’ via community education, information and awareness of disability and services.

The multiple roadblocks described throughout carers’ journeys are significant barriers to accessing services and support. The impact of transport is a particularly important consideration and is reflected in other studies of service access [51, 54, 55]. For carers, services that provided transport to and from appointments were invaluable and facilitated service access. Having to wait months to access services and confusion caused by interaction with multiple service providers has also been found in an Australian study of the experiences of CALD carers accessing developmental assessment services [49] suggesting the widespread nature of these barriers. Roadblocks are particularly concerning as accessing assessment services to obtain a diagnosis is essential in being able to access support and services [54]. Removing those barriers may help patients seek and access the medical care they need. Dedicated patient navigators may be a solution to overcome these barriers by bridging the gap between carers and the various service systems involved [55]. A patient navigator is akin to a tour guide who assists patients to navigate their way through complex systems, helping to remove patient-level barriers to reduce delays in accessing services [56, 57]. The patient navigator model can be a key ‘weapon’ against health disparities that certain groups face [56, 58]. A study of the experiences of Aboriginal patients with cancer accessing diagnosis and treatment found that a patient navigator model would help facilitate mainstream service access for patients [59]. Involving Aboriginal liaison officers in these roles is a way to foster cultural security [60]. Focus groups with key stakeholders and Aboriginal carers have also identified that patient navigators in the form of dedicated disability support workers within Aboriginal health services would help in raising awareness of available support and services for families [21]. While a patient navigator could help carers to by-pass roadblocks at the service level, responsive service systems are critical to the model’s efficacy. Responsive services systems that are informed by the views of both carers and the workforces responsible for service delivery, especially the Aboriginal and Torres Strait Islander workforce, are important to understanding the needs of carers and developing responsive strategies to address them [61]. Guiding principles for responsive service systems include sharing ownership and responsibility for change, responding to the needs of families in the context of where they live, as well as the Aboriginal and Torres Strait Islander workforce, working together from the micro to macro levels, and building on the existing strengths of families and relevant workforces [61].

The use of alternate routes or service models is important. Future planning of service and support models should consider the elements identified by carers as most important including a holistic view of the needs of the child and carer, one-stop-shop for services and operationalizing a centralized team-based approach. These elements are typically embodied in the model of care provided by ACCHOs and argued to be a vital part of improving health and wellbeing outcomes for Aboriginal and Torres Strait Islander peoples [62].

The incompatibility between inflexible bureaucratic policy requirements and the demands and challenges of caring for a child with a disability was a significant part of carer journeys. Meeting inflexible requirements for eligibility to respite services and funding has elsewhere been described as ‘jumping through hoops’ [54]. Given the multiple complex needs of these families and frequent interaction with government departments, Butler et al. [55] recommend that modification of inflexible policy requirements is essential if design of policies and programs are to improve service access for parents in vulnerable families [55].

### Limitations

Participants were purposefully sampled in order to facilitate in-depth exploration of their experiences. However, self-selection bias along with a small sample size means that these findings are not necessarily generalizable to other Aboriginal and Torres Strait Islander populations or to indigenous populations globally. All data were self-reported reflecting carers’ individual experiences and perspectives. Further research into the experiences of service providers would help contribute to a more comprehensive picture of barriers and facilitators to service access. Follow-up interviews were conducted within a 12 month period despite more prolonged service access journeys. All participants in this study were women, hence the perspectives of male carers is needed.

## Conclusions

The challenges of caring for a child with a disability are indisputable. The socio-economic challenges and marginalisation experienced by many Aboriginal and Torres Strait Islander people compound these challenges. Building community awareness of disability services and support, increasing access to alternate routes to care, and consideration of roadblocks and disadvantageous road rules in service delivery as well as ways of overcoming them are important to facilitating service access.

## Additional file

Additional file 1: Topic guide for interview with parent/carer. (DOCX 13 kb)

## Abbreviations

ACCHOs: Aboriginal Community Controlled Health Organisations
AEOs: Aboriginal education officers
AH&MRC: Aboriginal Health and Medical Research Council
CALD: Culturally and linguistically diverse
DoH: Department of Housing
GP: General practitioner
OT: Occupational therapist
