# Supplementary material for: Navigating the journey of Aboriginal childhood disability: a qualitative study of carers’ interface with services
Source: BMC Health Serv Res. 2016 Dec 1;16:680. doi: 10.1186/s12913-016-1926-0 (PMC5134075; doi:10.1186/s12913-016-1926-0)
Supplement: Additional file 1: — Topic guide for interview with parent/carer. (DOCX 13 kb) [file 12913_2016_1926_MOESM1_ESM.docx]

**Topic guide for interview with parent/carer**

**Background and contextual information**

Tell us about your situation in regards to caring for (child)

How has this affected your life?

(Ascertain family members, ages, health conditions, employment status/caregiving responsibilities)

**Navigating health and social services systems**

Pathway to support/treatment - How do you get information and services you need?

Interaction with health professionals, services, facilities

Adequate timing of available support/wait list?

**Affordability**

Receiving financial support from Centrelink? Other?

Perception of adequacy/need

**Accessibility**

Travel/mobility/transport issues

**Impact on carer/family/child**

Mental and physical health and wellbeing

Sources of support

Community

**Barriers**

What has made it hard to get care for (child)?

**Facilitators**

Has anything been helpful?

Can you think of what might make your life/caring for (child) easier? / Preference for support model?

**Respite experiences**

**Future outlook**
